# Complete Genome Sequence of Klebsiella pneumoniae Strain TK421, a Conjugative Hypervirulent Isolate

**DOI:** 10.1128/MRA.01408-19

**Published:** 2020-01-16

**Authors:** Travis J. Kochan, Egon A. Ozer, Nathan B. Pincus, Margaret A. Fitzpatrick, Alan R. Hauser

**Affiliations:** a Department of Microbiology-Immunology, Northwestern University Feinberg School of Medicine, Chicago, Illinois, USA; b Department of Medicine, Division of Infectious Diseases, Northwestern University Feinberg School of Medicine, Chicago, Illinois, USA; c Department of Medicine, Division of Infectious Diseases, Loyola University Chicago Stritch School of Medicine, Maywood, Illinois, USA; University of Arizona

## Abstract

Klebsiella pneumoniae is a Gram-negative bacterium that is a major cause of nosocomial infections worldwide. Here, we present the complete genome sequence of TK421, a clinical bacteremia isolate containing a hypervirulence plasmid carrying *tra*-associated conjugation machinery genes. Emergence of conjugative hypervirulence plasmids could portend rapid dissemination of hypervirulence among multidrug-resistant K. pneumoniae strains.

## ANNOUNCEMENT

Klebsiella pneumoniae is a Gram-negative pathogen that is a major cause of nosocomial infections worldwide (1–3). K. pneumoniae is a commonly multidrug-resistant (MDR) bacterium and a frequent cause of serious infections in immunocompromised patients residing in hospitals and long-term-care facilities (3–8). Some K. pneumoniae isolates manifest as invasive community-acquired infections known as hypervirulent K. pneumoniae (hvKP) infections (9–12). hvKP isolates are defined by the presence of several biomarkers commonly associated with a large virulence plasmid comprising aerobactin and salmochelin biosynthesis genes and the capsule mucoid regulators *rmpA* and *rmpA2* (13). While hvKP strains cause serious infections in immunocompetent patients, they are typically antimicrobial susceptible. A major public health concern is convergence of hvKP and MDR K. pneumoniae to produce highly virulent strains that are difficult to treat with antimicrobial agents (14–16).

In this study, we determined the complete genome sequence of hypervirulent Klebsiella pneumoniae strain TK421, a clinical bacteremia isolate collected from Northwestern Medical Center in Chicago, Illinois, on 3 September 2013. After detection of a positive blood culture, single colonies were isolated on 5% sheep blood agar. Genomic DNA was extracted using the Maxwell 16 system (Promega Corp., Madison, WI) from a single colony inoculated into lysogeny broth and cultivated at 37°C overnight with shaking. The SMRTbell template preparation kit v1.0 (Pacific Biosciences, Menlo Park, CA) was used to ligate hairpin adapters required for sequencing sheared genomic DNA. Libraries were sequenced using PacBio P6-C4 chemistry and RS II single-molecule real-time (SMRT) cells 8Pac v3 with 240-min movies on an RS II instrument (Pacific Biosciences). PacBio sequencing yielded 206,462 reads (mean length, 13,095 bases) totaling 2.70 Gb of sequence for an approximate genome coverage of 509-fold. These reads had an *N*_50_ value of 22,681 bases and an *L*_50_ value of 42,692 bases. Libraries for Illumina sequencing were prepared using the Nextera XT library kit (Illumina, Inc., San Diego, CA) and sequenced on an Illumina MiSeq instrument with a V3 flow cell to generate paired-end 301-bp reads, yielding 705,712 reads totaling 194 Mb of sequence for an approximate genome coverage of 35-fold. Hybrid assembly and circularization of PacBio and Illumina reads were performed with Unicycler v0.4.8 (17). Annotation was performed using the NCBI Prokaryotic Genome Annotation Pipeline (18, 19). Kleborate analysis was used to determine sequence type and capsule type (20).

The final assembly of TK421 consisted of one circularized chromosome sequence of 5,277,246 bp and three circularized plasmid sequences of 130,329 bp, 127,353 bp, and 30,122 bp (pTK421_1 to pTK421_3, respectively). TK421 is closely related to sequence type 34 (ST34) but has two single-nucleotide variants (SNVs) in the *infB* allele. Sequence analysis suggested that it contains several virulence genes found on both the chromosome and mobile elements (20). The chromosome contains genes predicted to encode yersiniabactin biosynthesis enzymes, type 3 fimbriae proteins, and a KL20-like capsule (21). Plasmid pTK421_1 is a 130-kb incFII plasmid. Plasmid pTK421_2 is a 127-kb virulence plasmid that contains the mucoid regulator (*rmpA*) and aerobactin and salmochelin biosynthesis genes similar to those found on known hypervirulence plasmids (pKP52.145, pLVPK, and pK2044) (22–25). In addition, pTK421_2 contains *tra* conjugation machinery genes (*traDFGHIS* and *trbB*) (26, 27). Plasmid pTK421_3 is a 30-kb plasmid related to pSer-840e, a plasmid isolated from *Serratia* sp. isolate SSNIH1 (28). This study, along with a recent report of a hypervirulence plasmid with conjugative machinery in Klebsiella variicola (29), highlights the risk of hypervirulence genes disseminating to MDR *Klebsiella* strains through plasmid-mediated conjugation.

### Data availability.

The Illumina and PacBio sequencing reads were deposited in the NCBI Sequence Read Archive (SRA) under accession number SRP227550. The whole-genome hybrid assembly has been deposited at DDBJ/ENA/GenBank under BioProject number PRJNA449090 with accession numbers CP045691 to CP045694 (GCA_009601745).
